# Microwave-Assisted Solvothermal Synthesis of Nanocrystallite-Derived Magnetite Spheres

**DOI:** 10.3390/ma15114008

**Published:** 2022-06-05

**Authors:** Greta Zambzickaite, Martynas Talaikis, Jorunas Dobilas, Voitech Stankevic, Audrius Drabavicius, Gediminas Niaura, Lina Mikoliunaite

**Affiliations:** 1 Department of Organic Chemistry, Center for Physical Sciences and Technology (FTMC), Sauletekio al. 3, 10257 Vilnius, Lithuania; greta.zambzickaite@ftmc.lt (G.Z.); martynas.talaikis@ftmc.lt (M.T.); 2 Department of Functional Materials and Electronics, Center for Physical Sciences and Technology (FTMC), Sauletekio al. 3, 10257 Vilnius, Lithuania; jorunas.dobilas@ftmc.lt (J.D.); voitech.stankevic@ftmc.lt (V.S.); 3 Department of Characterization of Materials Structure, Center for Physical Sciences and Technology (FTMC), Sauletekio al. 3, 10257 Vilnius, Lithuania; audrius.drabavicius@ftmc.lt; 4 Department of Physical Chemistry, Faculty of Chemistry and Geosciences, Vilnius University, Naugarduko st. 24, 03225 Vilnius, Lithuania

**Keywords:** magnetite, Fe_3_O_4_, microwave-assisted solvothermal method

## Abstract

The synthesis of magnetic particles triggers the interest of many scientists due to their relevant properties and wide range of applications in the catalysis, nanomedicine, biosensing and magnetic separation fields. A fast synthesis of iron oxide magnetic particles using an eco-friendly and facile microwave-assisted solvothermal method is presented in this study. Submicron Fe_3_O_4_ spheres were prepared using FeCl_3_ as an iron source, ethylene glycol as a solvent and reductor and sodium acetate as a precipitating and nucleating agent. The influence of the presence of polyethylene glycol as an additional reductor and heat absorbent was also evaluated. We reduce the synthesis time to 1 min by increasing the reaction temperature using the microwave-assisted solvothermal synthesis method under pressure or by adding PEG at lower temperatures. The obtained magnetite spheres are 200–300 nm in size and are composed of 10–30 nm sized crystallites. The synthesized particles were investigated using the XRD, TGA, pulsed-field magnetometry, Raman and FTIR methods. It was determined that adding PEG results in spheres with mixed magnetite and maghemite compositions, and the synthesis time increases the size of the crystallites. The presented results provide insights into the microwave-assisted solvothermal synthesis method and ensure a fast route to obtaining spherical magnetic particles composed of different sized nanocrystallites.

## 1. Introduction

Magnetic particles are highly desirable in many scientific fields, especially biomedical fields [1,2], starting from MRI contrast agents [3,4], drug delivery systems [5,6], magnetic separators [7] and hyperthermia agents [8] and followed by the environmental [9], catalysis [10] and biosensing [11,12] fields. These nanoparticles capped with a plasmonic silver or gold layer could also be applied in surface-enhanced Raman spectroscopy due to signal enhancement for two reasons: the concentration of the sample using magnet and surface plasmons [13,14,15]. Magnetite particles are suitable for such applications due to their stability, biocompatibility, uncomplicated synthesis, low price and great magnetic response. However, the development of synthesis methods is still extremely important and not completely clear in obtaining a tailor-made product.

Many synthesis routs can be applied to obtain magnetic particles. They can be divided into physical, biological and chemical methods [2]. The physical methods are usually called top-down methods, where bulk material is shredded to smaller pieces by ball milling, laser evaporation or any other physical method. The biological synthesis routes employ biological objects that, in the presence of Fe ions in a solution, could produce nanoparticles within their structure or in the solution. Chemical synthesis, otherwise known as the bottom-up method, is the most widely applied in the synthesis of magnetic structures. This route could be further divided according to the synthesis procedure: hydrothermal [16], solvothermal [12,17], thermal decomposition [18], microwave-assisted [19,20], sol-gel [21,22], coprecipitation [23] and others [4]. In addition to this, the combination of two methods could be used: microwave-assisted solvothermal [24,25,26,27,28,29] or hydrothermal [20,30] methods or microwave assisted thermal decomposition methods [31].

Microwave synthesis is now gaining popularity due to its simplicity and reduced processing time. Microwave heating, which is mainly described as heat obtained from transformed electromagnetic energy, is used to accelerate the synthesis procedure instead of conventional heating. If the latter is used, a sample is heated starting from the sides of the reaction vessel due to the conduction and convection processes; thus, the temperature gradient appears, which causes non-uniform particle nucleation and growing processes. Microwave heating does not pose such a problem, since the energy is transferred through the sample volume (or microwave absorbing material) instantly and the heating is homogeneous [20]. During microwave heating, two effects can be distinguished: thermal and non-thermal. The first one is considered as fast and homogeneous and thus as an effective heating method of the sample volume, resulting in nucleation and growth. This gives uniform and high crystallinity results. During the non-thermal effect, hot spots and hot surfaces are created when heating solid surfaces at the solid–liquid interface. This process also supports the reduction of precursors, nucleation and the formation of particles [19]. In the microwave synthesis route, both organic and inorganic media could be used, and the synthesis time could be reduced to minutes instead of hours or days. Therefore, it is an energy-saving method as well. The microwave-assisted solvothermal method provides the possibility to reduce the synthesis time efficiently [26,27]. In addition, the modification of nanoparticles by coating with 3-(trimethoxysilyl)-1-propanethiol (TMSPT) and the subsequent modification by coating with 2-amino-5-mercapto-1,3,4-thiadiazole (AMP) to increase the stability of the nanoparticles are possible through this technique [26]. Thus, the whole process, including the synthesis of the nanoparticles, the coating with TMSPT and the modification with AMP, was accomplished during a short period of time (30 min) [26]. Furthermore, the semicrystalline Fe_3_O_4_ nanoparticles with an average diameter of 15 nm generated by the microwave-assisted solvothermal process were demonstrated to be active in the photocatalytic degradation of azo dye methyl orange and tetracycline under visible light radiation [27].

The medium of the synthesis varies from the most common, water, to organic solvent (e.g., glycol). All of these conditions severely affect the resulting material properties. Particles of various shapes, sizes, crystal structures and magnetization extents could be obtained. The shapes vary from cubic to spherical or rodlike [32]. Sizes from a few nanometers to several hundred nanometers could be achieved [33,34]. A few iron oxide crystal structures are known: the hematite (α-Fe_2_O_3_)—rhombohedral or hexagonal; the maghemite (γ-Fe_2_O_3_)—cubic or tetrahedral; and the magnetite (Fe_3_O_4_ or FeO·Fe_2_O_3_)—cubic [35]. They are most commonly found in nature, soil and rocks from volcanic eruptions, as well as from air pollution (emissions from traffic and industries) and from being synthesized in the laboratory.

Another highly important compound is the reducing agent (NaOH, ammonia and H_2_O_2_ are the most popular in aqueous solutions or glycol in nonaqueous synthesis) [6,15,23,33,36,37]. Finally, a stabilizing/capping agent may also be used. This could be some surfactant (for electrostatic stabilization such as sodium oleate, dodecylamine and sodium carboxymethyl cellulose) or polymer (for steric and, in some cases, electrostatic stabilization—for example, the chemical polymers polyethylene glycol, poly(vinyl alcohol), poly(lactic-co-glycolic acid), poly(vinyl-pyrrolidone), poly(ethylene-co-vinyl acetate) and other polymers [38], or natural polymer systems including gelatin, dextran and chitosan [39].

Polyethylene glycol is a biocompatible, hydrophilic, water-soluble organic polymer. Due to its high polarizability, it is an excellent microwave-absorbing agent that ensures a high heating rate and a significantly shorter reaction time. Harraz and colleagues synthesized single-phase magnetic nanowires using PEG in aqueous media and noticed that the amount of PEG in the reaction solution affects the size and crystallinity of the obtained results [40]. The porosity of the obtained nanoparticles during the microwave-assisted solvothermal synthesis was evaluated by Juang et al. [24].

In this work, organic magnetite synthesis in a microwave reactor is investigated. A combination of two methods, solvothermal and microwave, was used, resulting in microwave-assisted solvothermal synthesis. The advantages of such synthesis route include the saving of time and energy. In addition to this, benignancy to the environment is also an advantage, since the process is carried out in a closed system and a strong acid/base was not used in the initial solutions. It is known that microwave radiation is a great source of energy that offers a clean and effective form of heating [24]. The reaction was conducted in ethylene glycol, which acted as a reducing agent, as well in the presence of acetate, which acted as a nucleating/capping agent [25]. Additionally, the impact of polyethylene glycol was evaluated. During the synthesis, spherical magnetic particles with different morphologies were obtained. The microwave synthesis conditions such as the temperature and time were investigated. The structural, morphological and magnetic characterizations of the obtained particles are presented. The novelty of our work consists in the development of a simple microwave-assisted solvothermal method for the synthesis of spherical magnetic Fe_3_O_4_ particles consisting of nanocrystallites that are 10−30 nm in size and a systematic investigation on the temperature, time and availability of PEG, resulting in a successful reaction. The obtained results could provide more insight into the microwave-assisted solvothermal synthesis, providing a very fast route to obtaining spherical magnetic submicron sized particles composed of different sized nanocrystallites with a magnetite or magnetite/maghemite mix structure and helping to analyze their physical and chemical properties.

## 2. Materials and Methods

### 2.1. Materials

Polyethylene glycol (PEG, MW 20000) and ethylene glycol (EG) were purchased from Carl Roth GmbH (Karlsruhe, Germany); sodium acetate (NaCH₃COO; NaOAc) was obtained from Alfa Aesar (Haverhill, MA, USA); and iron(III) chloride (FeCl_3_) was obtained from Sigma Aldrich (St. Louis, MO, USA). All of the chemicals were of analytical grade and were used as obtained.

### 2.2. Synthesis of Magnetic Fe_3_O_4_ Particles

**Figure 1:**
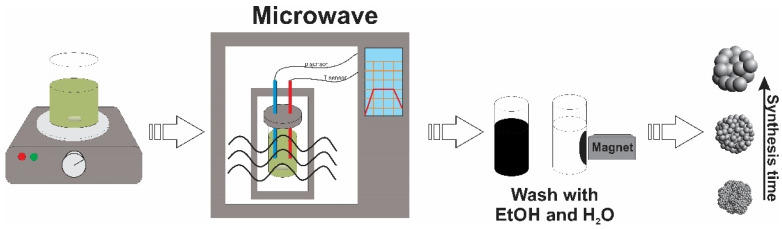
Synthesis scheme of Fe_3_O_4_ submicron spheres.

The synthesis of magnetite (Figure 1) was adopted from [24]. Firstly, 0.003 mol of iron(III) chloride was dissolved into 20 mL of ethylene glycol in a 150 mL glass beaker. Magnetic stirring at 50 °C for 10 min was applied. Sodium acetate (0.0122 mol) and PEG (0.5 g optional) were added into the solution under vigorous stirring (50 °C, 500 rpm), and the conditions were maintained until the materials were completely dissolved and the color of the solution became dirty yellow. A well-mixed solution was put into the microwave reactor (flexiWAVE, Milestone Srl, Milan, Italy), which was performing under 2.45 GHz of microwave irradiation. Temperature control was ensured by an optical fiber thermal sensor inserted into the glass tube with the reaction mixture. The tube was placed in a well-sealed Teflon vessel to maintain the pressure during the heating process. The reactor provided uniform sample heating to support the reaction, while the ethylene glycol with a higher dielectric constant was used as an energy adsorption agent. Continuous stirring and various times (from 1 to 120 min) and temperatures (200–250 °C) were used during the synthesis procedure. The temperature raising time was set to 5 (for a longer synthesis) or 2 (for a shorter synthesis) minutes. The longer time was chosen due to the higher stability of the temperature flux; however, when the synthesis time was shorter than 5 min, the temperature raising time was also reduced. After the synthesis, the solution became black, and the magnetic precipitates were collected using a neodymium magnet, washed with ethanol (three times) and distilled water (one time) and left to dry in an oven (120/300 LSN 11, SNOL, Utena, Lithuania).

### 2.3. Characterization of Magnetic Fe_3_O_4_ Particles

The structures of the obtained magnetic particles were characterized using: XRD, Raman and infrared absorption spectroscopy, TEM and TGA. The magnetization of the particles was measured in a pulsed magnetic field by an induction method using well-compensated pick-up coils. For the characterization of the crystalline structure of the particles, XRD measurements were conducted using a MiniFlex (Rigaku, Tokyo, Japan) X-ray diffractometer with a scanning region of 2Θ from 10° to 80°. The morphologies of the samples were studied using a high-resolution transmission electron microscope (HR-TEM Tecnai G2 F20 X-TWIN, FEI, Hillsboro, OR, USA). The infrared absorption (FTIR) spectra were recorded from particles dispersed in the KBr pellets using an Alpha spectrometer (Bruker, Inc., Karlsruhe, Germany) equipped with a room temperature RT-DLATGS detector. Some of the samples were measured by using the ATR accessory (Platinum ATR Diamond, Karlsruhe, Bruker). All of the FTIR spectra were collected with 4 cm^−1^ resolution from 20 scans. The Raman spectra of the particles were obtained by an inVia Raman microscope (Renishaw, Wotton-under Edge, UK) equipped with a CCD camera thermoelectrically cooled to −70 °C. The 830 nm laser excitation was restricted to 0.8 mW and was focused on the sample by a 20×/0.4 NA objective lens to a line-shape area on a sample of approximately 10 μm × 90 μm. The Raman signal was dispersed by using 830 lines/mm grating. Each sample was recorded at three different spots on its surface, with a 20 min acquisition time. The Raman wavenumber axis was calibrated by using the silicon standard peak at 520.7 cm^−1^. The thermogravimetric analysis was performed using the STA6000 (Perkin Elmer, Waltham, MA, USA) with a 0.18 mL aluminum oxide crucible. The temperature was raised from 22 °C to 500 °C in a nitrogen atmosphere (20 mL/min). The temperature rising step was set to 10 °C/min. The magnetic characteristics at room temperature (294 K) were measured using pulsed-field magnetometry.

## 3. Results and Discussion

### 3.1. Investigation of Synthesis of Magnetic Particles

**Figure 2:**
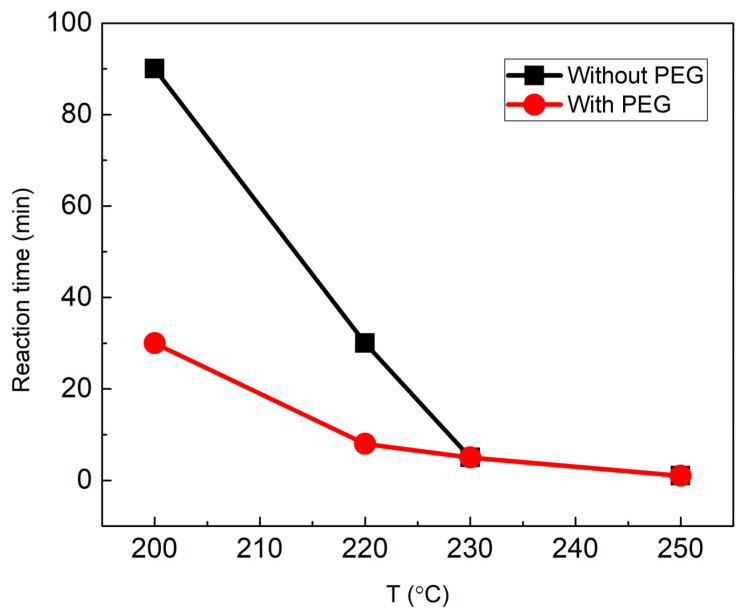
Reaction time dependence on the temperature maintained by the microwave reactor. A difference is noticed in the reaction times at low temperatures when PEG is added to the initial reaction mixture.

The syntheses of the magnetic particles (MPs) were performed at different time and temperature values. All of the used combinations are presented in Table 1. The temperature scale was set from 200° to 250 °C. A temperature of 180 °C was also examined; however, even after 2 h of synthesis, black magnetic precipitates were still not visible. This might be due to the boiling point of ethylene glycol, which is 197 °C. Only the temperatures above the EG boiling point induce the reduction of Fe ions. It was noticed that various temperatures above the boiling point accelerate the particle formation reaction differently. For example, at 200 °C, the synthesis of MPs (without PEG in the synthesis mixture) results in black precipitates only after 90 min. If the temperature is raised to 250 °C, particles are formed even after 1 min in the microwave reactor (additionally, 2 min of rising temperature was set). The influence of the addition of polyethylene glycol (PEG) was also investigated. PEG is known to be a reductor for silver nanoparticles [41], so the reaction time of the formed Fe_3_O_4_ particles should also be affected. As could be seen in Table 1, this is true at low temperatures. At 200 °C, the reaction is completed after 30 min instead of 90 min without PEG. At 220 °C, with PEG, particles are obtained in 8 min, while without PEG, this would take 30 min. For a better understanding, a graph comparing the successful syntheses in the shortest time with and without PEG is presented (Figure 2). The influence of PEG is visible at low temperatures: 200–220 °C. However, at high temperatures (230–250 °C), no difference can be noticed, and the particles are obtained in a short (1–5 min) interval.

### 3.2. X-ray Diffraction Patterns

**Figure 3:**
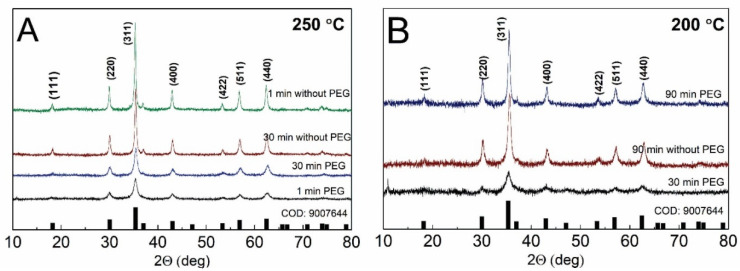
XRD diffractograms of Fe_3_O_4_ particles at synthesis temperatures of 250 °C (**A**) and 200 °C (**B**). The diffractograms of different synthesis times are presented, as well as a presence of PEG in the reaction mixture.

The crystal structure and phase purity of the obtained MPs were evaluated using XRD. The obtained patterns are shown in Figure 3A,B. All the obtained spectra were similar and matched the cubic Fe_3_O_4_ phase. The main obtained peaks—(220), (311), (400), (422), (511) and (440)—at 30.1°, 35.5°, 43.2°, 53.5°, 57.1° and 62.8° 2Θ values, respectively, match the COD (Crystallography Open Database) file, No. 9007644. At the most intense spectra, lower intensity peaks are also visible.

The intensity and the full width at half maximum (FWHM) of the diffraction peaks of the samples differ as well. In Figure 3A, the diffractograms of the particles synthesized at 250 °C are presented. For comparison, the syntheses with PEG and without PEG at synthesis times of 1 min and 30 min were chosen. All of them follow the XRD pattern for the magnetite structure; however, the intensity and FWHM of the spectra differ. Without PEG, the synthesized particles show higher intensity XRD spectra, while both spectra with PEG show lower intensity. It is known that the intensity or FWHM of the XRD peaks could be associated with the crystallite size. If the crystal structure is large, the obtained peaks are of a higher intensity, and small crystallites could show the low intensity of the XRD signal. From these data, particles with PEG are expected to be smaller in comparison to particles synthesized for the same time but without PEG.

The XRD diffractograms were also compared for the samples synthesized at 200 °C. Figure 3B compares the particles obtained at the shortest possible synthesis times: 30 min with PEG and 90 min without PEG. The 90 min PEG-synthesis is also added for comparison. The diffractograms obtained at the same time—90 min—were of similar intensities; however, the intensity of the one obtained after 30 min of synthesis was the lowest. This, as well as the previous section, indicates that shorter synthesis times yield particles of a smaller crystallite structure, and, in time, the crystallites are growing, resulting in a more intensive XRD signal.

### 3.3. Raman Spectra Analysis

**Figure 4:**
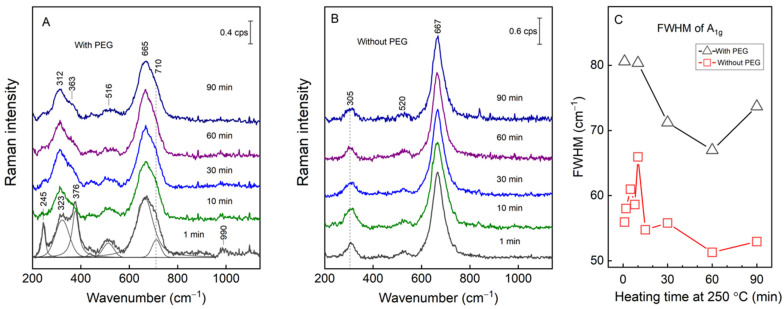
Raman spectra of the samples prepared with (**A**) and without (**B**) PEG heated from 90 to 1 min. The dependence of the full width at half maximum (FWHM) of the A_1g_ mode on the MPs preparation time at 250 °C with (black triangles) and without (red squares) PEG (**C**). The excitation wavelength was 830 nm; the laser power was set to 0.8 mW; the acquisition time was 60–120 min.

Magnetite and maghemite having the same spinel structure and almost identical lattice parameters make the identification of magnetite and maghemite by the XRD technique complicated [25,42]. However, Kozakova et al. suggested using the (511) Bragg peak (in the range of 56.5–57.5 of 2Θ) as an identification tool. For pure magnetite, the central position of the diffraction peak should be at 57.0°, while for maghemite, this peak is slightly shifted to higher values, i.e., 57.3° [25]. In our case the peak has a maximum at 57.1°, suggesting that the main structure of the sample is magnetite with some amount of maghemite. To analyze the crystal structure of magnetite and detect the possible secondary phases, Raman spectra were recorded. The literature suggests five Raman active vibrational modes at 193 cm^–1^ (T_2g_), 306 cm^–1^ (E_g_), 450–490 cm^–1^ (T_2g_), 538 cm^–1^ (T_2g_) and 668 cm^–1^ (A_1g_) [43]. The characteristic peaks of magnetite in our measurements could be seen at 663–668 cm^–1^, 308–310 cm^–1^ and close to 520 cm^–1^ for the samples prepared with and without PEG (Figure 4A,B). Raman spectra were recorded for the microwave heated samples from 1 to 90 min at 250 °C. The laser power density was reduced to 0.1 kW/cm^2^ at the expense of longer acquisition times to ensure that no laser-induced photolytic and pyrolytic effects would take place in the sample [44,45]. Contrary to the synthesis without PEG, the 1 min microwave preparation at 250 °C with PEG resulted in MPs with distinctive narrow-bandwidth spectral modes at 245 and 376 cm^–1^ (Figure 4B). These low-wavenumber bands are associated with other secondary phases, most likely goethite and lepidocrocite [46], which were no longer present in the Raman spectra at increasingly longer heating times. The A_1g_ mode’s asymmetry hinted at the presence of maghemite. Indeed, after fitting the experimental spectrum with Gaussian–Lorentzian shape components, the mode at 710 cm^–1^ was identified, which was directly associated with maghemite’s A_1g_ mode. We estimated a 12–16% contribution to the total integral intensity from the maghemite at each tested microwave preparation with PEG. A stark difference can be seen in the A_1g_ mode’s bandwidth expressed as the FWHM when the samples prepared with and without PEG are compared (Figure 4C). Generally, the FWHM correlates with the crystal structure of the sample and decreases with increasing crystallinity. It is well-known that the spectral modes of magnetite are much broader compared to, for example, those of hematite, due to the strong electron–phonon interactions [44,47]. However, the PEG preparation resulted in particles with bandwidths that were larger by 18 cm^–1^ on average compared to the ones prepared without PEG but with the same microwave heating time. The XRD data already confirmed larger crystallites in the nanoparticles prepared without PEG. A more quantitative analysis of the Raman bandwidths of the A_1g_ mode is provided in Figure 4C, where the FWHM is plotted against the sample preparation time at 250 °C. Our data show that, during the first 10 min of preparation without PEG, the FWHM increased from 56 to 66 cm^–1^, which was followed by a sharp drop to 55 cm^–1^ at 15 min, with no significant change in subsequent heating. For the PEG preparation of the MPs, a change from 80 to 71 cm^–1^ was detected for the first 30 min; later on, the changes were marginal. For both preparations, the heating up to 30 min decreased the FWHM by ca. 10 cm^−1^, indicating the growth of MPs with the increase in crystallinity in the samples with and without PEG.

### 3.4. TEM Images Analysis

**Figure 5:**
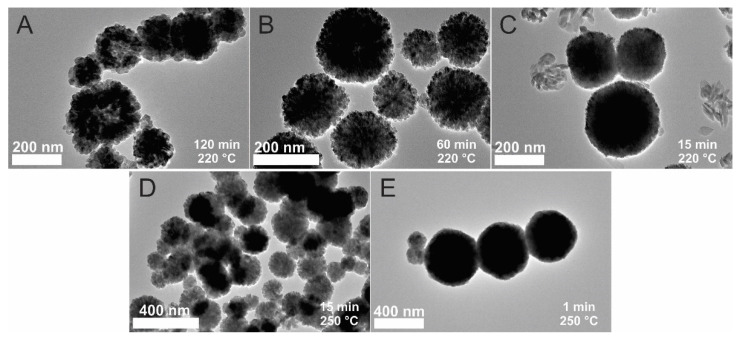
TEM images of the synthesized samples without PEG: 220 °C, 120 min (**A**); 220 °C, 60 min (**B**); 220 °C, 15 min (**C**); 250 °C, 15 min (**D**); 250 °C, 1 min (**E**). The scale bar for (**A**–**C**) is 100 nm, and the scale bar for (**D**) and (**E**) is 200 nm.

To confirm the different sizes of the crystallites, TEM images of the particles were captured. In Figure 5, the MPs obtained using synthesis without PEG at 220 °C and 250 °C are presented. These particles were synthesized at 220 °C for 120 (Figure 5A), 60 (Figure 5B) and 15 min (Figure 5C). Although the sizes of the particles are similar (around 200 nm (Table 2)), the structures of the MPs are quite different. The longest synthesized MPs are composed of larger sized crystallites (26 ± 4 nm). The synthesis that was 60 min in duration resulted in smaller crystallites (12 ± 2 nm), and the particles obtained after the shortest time of synthesis (15 min) had the smallest crystallites, the size of which was impossible to measure using the Image J program. The same results were also observed for the syntheses at higher temperatures. In Figure 5, the TEM images of the particles synthesized at 250 °C for 1 (Figure 5E) and 15 min (Figure 5D) are presented. The sizes of the spheres were more or less the same as those for the synthesis at 220 °C. Larger crystallites (25 ± 7 nm) were observed at longer synthesis times (15 min), while no observable crystallites were seen after 1 min of synthesis. The MP and crystallite sizes calculated by the Image J program are summarized in Table 2.

### 3.5. FTIR Spectra Analysis

**Figure 6:**
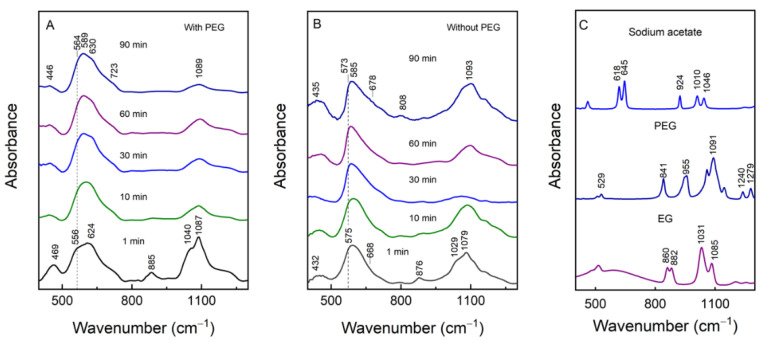
Preparation time at 250 °C-dependent FTIR spectra of KBr dispersed particles that were produced with (**A**) and without (**B**) PEG. The ATR-FTIR spectra of sodium acetate, PEG and ethylene glycol (EG) (**C**). The spectral positions in (**A**) and (**B**) were worked out from the second derivative spectrum calculated using the Savitzky–Golay algorithm.

To evaluate the presence of organic reductors on the MPs, FTIR spectra were recorded for the particles prepared with and without PEG (Figure 6). Magnetite, due to its spinal structure, has four infrared-active bands which appear at ca. 570 (ν_1_), 390 (ν_2_), 270 (ν_3_) and 180 (ν_4_) cm^–1^ [48,49,50,51]. The strong ν_1_ mode assigned to the Fe–O stretching motion of the tetrahedral and octahedral sites, when narrow, suggests the high crystallinity of the sample. In the case of the formation of defects and secondary phases, the modes become broader and shift. For maghemite, which is considered to be a defective form of magnetite, the Fe–O stretch absorption modes are expected at 630, 590 and 430 cm^–1^ [48,52]. We already discussed the relatively small contribution from γ-Fe_2_O_3_ to the particles prepared with PEG and the nonexistent contribution for the MPs prepared without PEG based on our Raman measurements. Therefore, the relatively broad infrared absorption feature near 600 cm^–1^ was ascribed to the Fe_3_O_4_ phase in nanoparticles of low crystallinity and, to some extent, to γ-Fe_2_O_3_. Notably, the preparation without PEG resulted in MPs with a somewhat narrower mode—near 600 cm^–1^. This is arguably due to the higher crystallinity of the MPs compared with the PEG MPs. The most obvious heating-time-dependent changes occurred within the first 10 min. These are especially visible in the 800–1100 cm^–1^ region, where the characteristic vibrations of the organic reductors could be seen. For example, the modes near 880, 1040 and 1080 cm^–1^ correspond to the vibrations of ethylene glycol and PEG (Figure 6C). Later, as the heating time passed the 10 min mark, the spectral changes in the PEG preparation were marginal, indicating that the reducing reagents had fully reacted and that no further changes had happened with the organic components in the reaction vessel.

### 3.6. Thermo Gavimetric Analysis

**Figure 7:**
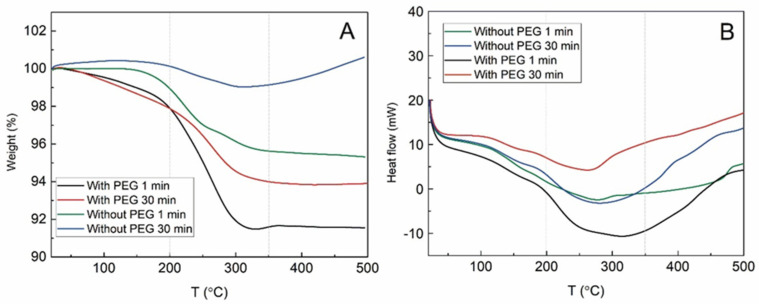
TGA data of samples synthesized with PEG and without PEG; synthesis time—1 or 30 min at 250 °C. Changes in sample weight (**A**) and heat flow (**B**) are registered from room temperature to 500 °C.

In Figure 7, the thermogravimetric analysis data of weight change and heat flow are presented. Few temperature intervals could be detected in the following samples: from RT to 200 °C, from 200 to 350 °C and from 350 to 500 °C. In the first interval, the endothermic loss of water and -OH groups are detected. The curve decrease is small, reaching up to 2% of the weight loss for the samples synthesized with PEG, while for the ones without PEG, the loss is 1% or even nonexistent. The second interval could be attributed to the desorption and subsequent evaporation of PEG or EG and the last interval—the phase transformation from Fe_3_O_4_ to γ-Fe_2_O_3_. The evaporation of PEG and EG resulted in a larger decrease in weight in comparison to that of the first interval. The samples with PEG lost 4–6% of their weight during the second interval, while the samples without PEG lost 1–4% of their weight. The highest amount of remnant organic material within the MPs prepared for 1 min was already demonstrated, as seen in the FTIR data. In the last interval, the weight of the samples remains similar—except for the sample synthesized without PEG for 30 min. Here, the increase of the weight at about 1% is registered. The phase transformation from Fe_3_O_4_ to γ-Fe_2_O_3_ is reached and then additional oxygen is introduced to the magnetite crystal structure. The process for the sample obtained after 30 min of synthesis without PEG is probably the most efficient, so the mass increase is registered. Although the mass of the sample stayed similar at the third interval, the heat flow was increasing, suggesting the occurrence of the exothermic process. The start of this process for all the samples begins at around 250–300 °C at the second interval. Possibly, the exothermic oxidation from Fe(II) to Fe(III) starts earlier and overlaps with the endothermic evaporation of the organics.

### 3.7. Investigation of Magnetic Properties

The magnetization *M* of particles was measured in a pulsed magnetic field with a duration of about 4 ms using a pulse magnetizer. The advantage of this method is that it allows for the rapid acquisition of isothermal magnetization, and a higher magnetic field can be applied to the samples. This method is widely used for measuring the magnetization of strong magnets [53], ferromagnetic materials or superconductors [54] and ferromagnetic powder or volcanic rock [55]. Moreover, it was demonstrated by Kodama [56] that the measurement of the magnetization of nanoparticles using this method is acceptable and yields results similar to those yielded by the vibration method. It was shown that a small difference in the obtained results is caused and phenomenologically explained by the difference in the time scale of the magnetization processes under consideration. That is, in the case of the pulsed field, a fraction of the magnetic particles with a relaxation time longer than the pulse rise time fails to follow the pulse. For the vibration method, the field sweep rate is about four orders of magnitude slower than the pulsed field duration, so most of the magnetic particles are magnetized simultaneously with the applied field.

In our work, all of the magnetic characteristics of the MPs using this method were compared at room temperature (294 K). The measurement system consists of a pulse magnetizer and two coils positioned inside of this magnetizer. The coils were connected with each other in opposite directions and were well-compensated. The coil of the pulse magnetizer with an inner diameter of 2.5 cm connected to the capacitor of 80 μF generates a pulsed magnetic field with an amplitude of 1.2 kOe and a pulse duration of 4 ms. The signal, directly proportional to *M* vs. time derivative, was obtained when the sample was placed in one of the coils and the capacitor was discharged through the pulse magnetizer. For the magnetic field measurements, the additional pick-up coil system was used. The saturations of mass magnetization (*M_S_*), coercivity (*H_C_*) and remanent magnetization (*M_r_*) were measured in this case.

**Figure 8:**
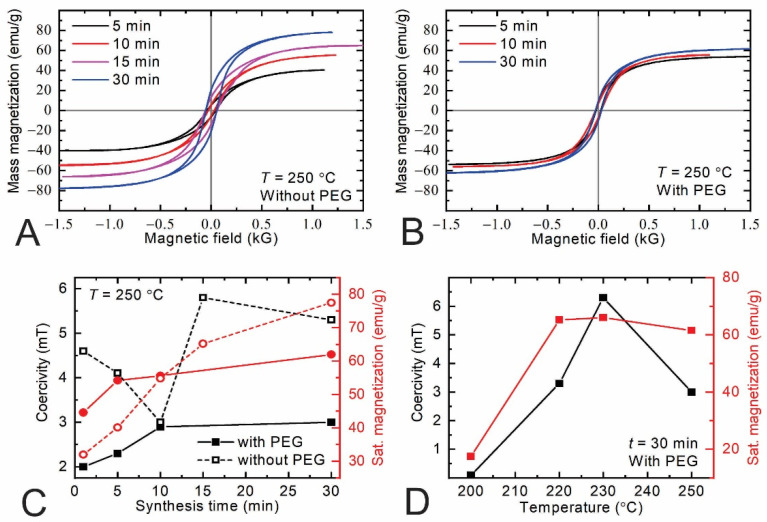
Hysteresis loop of the samples synthesized without PEG at 250 °C for different synthesis times—5, 10, 15 and 30 min—(**A**) and that of the samples synthesized with PEG (**B**). Magnetization (black symbols) and coercivity (red symbols) of the samples synthesized with PEG (solid symbols) and without PEG (empty symbols) using different times, from 1 to 30 min at 250 °C (**C**). Saturation magnetization (red symbols) and coercivity (black symbols) of the samples synthesized with PEG at different temperatures (**D**).

The nonlinear magnetization curves with the hysteresis loop, characteristic of the ferromagnetic behavior, are clearly observed in all the samples (Figure 8A,B). It was found, that, for the samples synthesized without PEG (see Figure 8A), the saturation magnetization *M_S_* increases with the increase in the synthesis time of the MPs. The magnetization of the particles that have been synthesized with PEG (Figure 8B) shows the same tendency, but the saturation value is lower than that for those that have been synthesized without PEG. The coercive field for all of the samples also depends on the synthesis method. The summarized results of *M_S_* and *H_C_* versus synthesis time are shown in Figure 8C. It can be seen that, for all of the samples, the increase in the synthesis time leads to an increase in the saturation magnetization and coercive field. For example, for the samples synthesized without PEG, the saturation magnetization increased from 32 emu/g to 78 emu/g when the synthesis time of the particles was changed from 1 min to 30 min. Meanwhile, the samples synthesized with PEG show a reduced value of *M_S_* for a longer times of synthesis, and it is changed from 46 emu/g to 62 emu/g, respectively. The lower magnetization can be attributed to the presence of a magnetically disordered layer or the existence of a secondary phase in these particles.

It is known that the magnetic properties of iron-oxide nanoparticles strongly depend on the particle size, shape and composition. Moreover, the synthesis method influences the stoichiometry of the nanoparticles, i.e., iron oxides can be synthesized in two main phases: magnetite (Fe_3_O_4_) or maghemite (γ-Fe_2_O_3_) [25,35,42]. Furthermore, it is known that MPs formed from magnetite have a much higher saturation magnetization than those formed from maghemite [57,58]. An analysis of the Raman spectra and data of the Fourier-transform infrared spectroscopy shows that the samples synthesized with PEG have a mixture of magnetite and maghemite, while for the samples without PEG, only magnetite is present. These results are also confirmed by the measurements of the particles’ magnetization. Moreover, the *M(H)* curve gives information about the domain structure in an ensemble of the MPs. It is well-known that magnetite nanoparticles that are smaller than 100 nm are in a single-domain state with a relatively low magnetization. An increase in particle size leads to a multidomain structure and an increased saturation magnetization [58,59,60]. Our results are in good agreement with the literature data. Table 2 shows that an increase in the synthesis time leads to an increase in the size of the crystallites, which are small grains in the submicron sized spheres.

The other magnetization parameter that was measured is the coercivity of the samples. It can be seen from Figure 8, that, for the samples prepared with PEG, the coercivity increases monotonously from 2 mT to 2.8 mT when the time of the synthesis is changed from 1 to 30 min, while for the samples prepared without PEG, it first decreases from 4.5 to 3 mT (until 10 min) and later increases up to 5.5 mT. These results could be explained by the peculiarity of the crystalline structures of MPs and also by the increase in their sizes. It was shown by Dehsari et al. [58] that the behavior of *H_C_* with the size is commonly attributed to the transition of the particle from a magnetically single-domain to a multidomain structure.

In addition, the obtained results of the magnetization of the samples were analyzed from the point of view of the dependence of their parameters on the synthesis temperature. The obtained results are shown in the Figure 8D. As can be seen from the graph, the magnetization and coercivity of the samples increase with the increase in synthesis temperature from 200 °C to 230 °C, but at higher temperatures, these parameters slightly decrease. However, this is related mostly to the peculiarities of the synthesis of the particles, not to their magnetic properties.

### 3.8. The Mechanism of Magnetite Formation

According to the literature, the microwave-assisted solvothermal synthesis of magnetite particles could be divided into two stages. The first is called the nucleation of primary crystals and the second is called the nanoparticles aggregation [27,61]. In the synthesis mixture, sodium acetate acts as a weak base, helping the EG to reduce iron ions, and, in the presence of the trace amount of water, it can be hydrolyzed and release OH^−^ ions. The trace amount of water could be obtained from the air as the initial mixture is stirred in an ambient atmosphere, and, in EG, about 0.5% (*w*/*w*) is water. Only the trace amount of water is required for the synthesis. The addition of extra water results in polyhedral particles of different sizes [25].

The hydrolysis of sodium acetate proceeds as follows (the net ionic equation is presented):

CH_3_COO^−^ + H_2_O ⇄ CH_3_COOH + OH^−^ (1)

The OH^−^ ions are consumed for Fe(III)hydroxide formation:

Fe^3+^ + 3OH^−^ ⇄ Fe(OH)_3_ (2)

which later may turn to Fe_2_O_3_:

2Fe(OH)_3_ ⇄ Fe_2_O_3_ + 3H_2_O (3)

In the meantime, ethylene glycol can undergo dehydration and form acetaldehyde [62]:

2HOCH_2_ − CH_2_OH ⇌ 2CH_3_CHO + 2H_2_O (4)

Then, the acetaldehyde reacts with Fe^3+^ ions and reduces them to Fe^2+^:

2CH_3_CHO + 2Fe^3+^ ⇌ CH_3_COCOCH_3_ + 2Fe^2+^ + 2H^+^ (5)

Additionally, an alternative pathway of EG may also exist. For example, the heating of EG in air may generate glycolaldehyde, a reductant for many metal ions [63]:

2HOCH_2_CH_2_OH + O_2_ ⇌ 2HOCH_2_CHO + 2H_2_O (6)

In any case, the obtained Fe^2+^ forms hydroxide:

Fe^2+^ + 2OH^−^ ⇌ Fe(OH)_2_ (7)

Finally, in the presence of both Fe ions, magnetite formation is enabled:

Fe(OH)_2_ + 2Fe(OH)_3_ ⇄ Fe_3_O_4_ + 4H_2_O (8)

The addition of microwaves is believed to facilitate hydroxide formation to oxide reaction (8), EG dehydration and subsequent reactions (due to the energy absorption and heating up of the solvent). In addition to this, secondary aggregation to the submicron size spheres is also believed to be caused by microwaves [27].

In the presence of PEG, more Fe_2_O_3_ crystalline structures are left. Possibly, the long molecules of PEG limit the diffusion of ions, and the first part of the reaction mechanism (1–3) is dominating. However, PEG is known to act as an additional reductor as well [41].

## 4. Conclusions

In this work, a facile and eco-friendly microwave-assisted solvothermal method is suggested for the synthesis of Fe_3_O_4_ magnetite spheres. Depending on the reaction temperature, the minimal time is suggested for fully synthesized MPs. At the temperature close to the ethylene glycol boiling point (200 °C), the shortest synthesis time is 90 min for the preparation without PEG and 30 min if PEG is used in the initial synthesis solution. However, when the temperature is increased to 250 °C, the fully synthesized magnetic particles are obtained even after 1 min of reaction (with an additional 2 min temperature raising time), independently of the presence of PEG. Although the sizes of the spheres at different synthesis times are similar, the crystal structures of these spheres differ. The longer the synthesis time is, the larger the obtained crystals are. These results were confirmed by TEM and XRD measurements. From the FTIR and Raman measurements, the sample synthesized with PEG contains a mixture of magnetite and maghemite, while for the samples without PEG, only magnetite is present. The magneticity measurement results also confirm this statement. It was found that the saturation magnetization and coercive field increase with the increase in synthesis time. We hope that this research will be beneficial for the further synthesis, development and applications of magnetite particles.

## Figures and Tables

**Table 1:** Microwave-assisted solvothermal synthesis of Fe_3_O_4_ particles with and without PEG in the synthesis mixture ^1^.

	Temp., °C	With PEG				Without PEG			
Time, min		200	220	230	250	200	220	230	250
1 ^2^					+				+
2 ^2^					+				+
5 ^2^				+	+			+	+
8			+	+	+			+	+
10			+	+	+			+	
15			+	+	+			+	+
30		+	+	+	+		+		+
45		+							
60		+					+		
75		+	+						
90		+			+	+			+
105		+							
120					+	+	+		+

^1^ Synthesis conducted at temperatures from 200 to 250 °C and from 1 to 120 min in the microwave reactor. The temperature rising time was set to 5 min unless stated otherwise. The successful reaction conditions (i.e., those that resulted in black magnetic precipitates) are marked with +. The table coloring indicates the conditions that yielded (green) and does not yield (blank) particles. ^2^ The temperature rising time is 2 min.

**Table 2:** Comparison of the Fe_3_O_4_ particle sizes synthesized without PEG at different temperatures and times. Particles refer to the whole sized spheres, and crystallites are the small grains of which the particles are comprised.

	220 °C			250 °C	
	15 min	60 min	120 min	1 min	15 min
Particles, nm	188 ± 27	182 ± 45	229 ± 55	303 ± 134	200 ± 29
Crystallites, nm	NA ^1^	12 ± 2	26 ± 4	NA ^1^	25 ± 7

^1^ Data is not available to obtain using ImageJ programe.

## Data Availability

The data presented in this study are available from the corresponding authors upon reasonable request.

## References

[1] Materón E.M., Miyazaki C.M., Carr O., Joshi N., Picciani P.H.S., Dalmaschio C.J., Davis F., Shimizu F.M. (2021). Magnetic Nanoparticles in Biomedical Applications: A Review. Appl. Surf. Sci. Adv..

[2] Ali A., Shah T., Ullah R., Zhou P., Guo M., Ovais M., Tan Z., Rui Y.K. (2021). Review on Recent Progress in Magnetic Nanoparticles: Synthesis, Characterization, and Diverse Applications. Front. Chem..

[3] Farzin A., Etesami S.A., Quint J., Memic A., Tamayol A. (2020). Magnetic Nanoparticles in Cancer Therapy and Diagnosis. Adv. Healthcare Mater..

[4] Reddy L.H., Arias J.L., Nicolas J., Couvreur P. (2012). Magnetic Nanoparticles: Design and Characterization, Toxicity and Biocompatibility, Pharmaceutical and Biomedical Applications. Chem. Rev..

[5] Dilnawaz F., Singh A., Mohanty C., Sahoo S.K. (2010). Dual Drug Loaded Superparamagnetic Iron Oxide Nanoparticles for Targeted Cancer Therapy. Biomaterials.

[6] Zhao Y., Qiu Z., Huang J. (2008). Preparation and Analysis of Fe3O4 Magnetic Nanoparticles Used as Targeted-Drug Carriers. Chin. J. Chem. Eng..

[7] Leong S.S., Ahmad Z., Low S.C., Camacho J., Faraudo J., Lim J.K. (2020). Unified View of Magnetic Nanoparticle Separation under Magnetophoresis. Langmuir.

[8] Liu X., Zhang Y., Wang Y., Zhu W., Li G., Ma X., Zhang Y., Chen S., Tiwari S., Shi K. (2020). Comprehensive Understanding of Magnetic Hyperthermia for Improving Antitumor Therapeutic Efficacy. Theranostics.

[9] Di S., Ning T., Yu J., Chen P., Yu H., Wang J., Yang H., Zhu S. (2020). Recent Advances and Applications of Magnetic Nanomaterials in Environmental Sample Analysis. Trends Analyt Chem..

[10] Sappino C., Primitivo L., de Angelis M., Domenici M.O., Mastrodonato A., Romdan I.B., Tatangelo C., Suber L., Pilloni L., Ricelli A. (2019). Functionalized Magnetic Nanoparticles as Catalysts for Enantioselective Henry Reaction. ACS Omega.

[11] Haun J.B., Yoon T.J., Lee H., Weissleder R. (2010). Magnetic Nanoparticle Biosensors. Wiley Interdiscip. Rev. Nanomed. Nanobiotechnol..

[12] Khorsand Zak A., Shirmahd H., Mohammadi S., Banihashemian S.M. (2020). Solvothermal Synthesis of Porous Fe_3_O_4_ Nanoparticles for Humidity Sensor Application. Mater. Res. Express.

[13] Lai H., Xu F., Wang L.A. (2018). Review of the Preparation and Application of Magnetic Nanoparticles for Surface-Enhanced Raman Scattering. J. Mater. Sci..

[14] Michałowska A., Krajczewski J., Kudelski A. (2022). Magnetic Iron Oxide Cores with Attached Gold Nanostructures Coated with a Layer of Silica: An Easily, Homogeneously Deposited New Nanomaterial for Surface-Enhanced Raman Scattering Measurements. Spectrochim. Acta A Mol. Biomol. Spectrosc..

[15] Michałowska A., Żygieło M., Kudelski A. (2021). Fe3O4-Protected Gold Nanoparticles: New Plasmonic-Magnetic Nanomaterial for Raman Analysis of Surfaces. Appl. Surf. Sci..

[16] Ge S., Shi X., Sun K., Li C., Uher C., Baker J.R., Banaszak Holl M.M., Orr B.G. (2009). Facile Hydrothermal Synthesis of Iron Oxide Nanoparticles with Tunable Magnetic Properties. J. Phys. Chem. C.

[17] Chen Y., Zhang J., Wang Z., Zhou Z. (2019). Solvothermal Synthesis of Size-Controlled Monodispersed Superparamagnetic Iron Oxide Nanoparticles. Appl. Sci..

[18] Unni M., Uhl A.M., Savliwala S., Savitzky B.H., Dhavalikar R., Garraud N., Arnold D.P., Kourkoutis L.F., Andrew J.S., Rinaldi C. (2017). Thermal Decomposition Synthesis of Iron Oxide Nanoparticles with Diminished Magnetic Dead Layer by Controlled Addition of Oxygen. ACS Nano.

[19] Tsuji M., Hashimoto M., Nishizawa Y., Kubokawa M., Tsuji T. (2005). Microwave-Assisted Synthesis of Metallic Nanostructures in Solution. Chem. Eur. J..

[20] Zhu X.H., Hang Q.M. (2013). Microscopical and Physical Characterization of Microwave and Microwave-Hydrothermal Synthesis Products. Micron.

[21] Xu J., Yang H., Fu W., Du K., Sui Y., Chen J., Zeng Y., Li M., Zou G. (2007). Preparation and Magnetic Properties of Magnetite Nanoparticles by Sol-Gel Method. J. Magn. Magn. Mater..

[22] Lemine O.M., Omri K., Zhang B., el Mir L., Sajieddine M., Alyamani A., Bououdina M. (2012). Sol-Gel Synthesis of 8 nm Magnetite (Fe3O4) Nanoparticles and Their Magnetic Properties. Superlattices Microstruct..

[23] Tai M.F., Lai C.W., Hamid S.B.A., Suppiah D.D., Lau K.S., Yehya W.A., Julkapli N.M., Lee W.H., Lim Y.S. (2014). Facile Synthesis of Magnetite Iron Oxide Nanoparticles via Precipitation Method at Different Reaction Temperatures. Mater. Res. Innov..

[24] Juang R.-S., Su C.-J., Wu M.-C., Lu H.-C., Wang S.-F., Sun A.-C. (2019). Fabrication of Magnetic Fe_3_O_4_ Nanoparticles with Unidirectional Extension Pattern by a Facile and Eco-Friendly Microwave-Assisted Solvothermal Method. J. Nanosci. Nanotechnol..

[25] Kozakova Z., Kuritka I., Kazantseva N.E., Babayan V., Pastorek M., Machovsky M., Bazant P., Saha P. (2015). The Formation Mechanism of Iron Oxide Nanoparticles within the Microwave-Assisted Solvothermal Synthesis and Its Correlation with the Structural and Magnetic Properties. Dalton Trans..

[26] Hernández-Hernández A.A., Álvarez-Romero G.A., Castañeda-Ovando A., Mendoza-Tolentino Y., Contreras-López E., Galán-Vidal C.A., Páez-Hernández M.E. (2018). Optimization of microwave-solvothermal synthesis of Fe_3_O_4_ nanoparticles. Coating, modification, and characterization. Mater. Chem. Phys..

[27] Zanchettin G., Falk G.S., González S.Y.G., Hotza D. (2021). High performance magnetically recoverable Fe_3_O_4_ nanocatalysts: Fast microwave synthesis and photo-fenton catalysis under visible-light. Chem. Eng. Process..

[28] Bhattacharjee S., Mazumder N., Mondal S., Panigrahi K., Banerjee A., Das D., Sarkar S., Roy D., Chattopadhyay K.K. (2020). Size-modulation of functionalized Fe_3_O_4_: Nanoscopic customization to devise resolute piezoelectric nanocomposites. Dalton Trans..

[29] Jing X., Liu T., Wang D., Liu J., Meng L. (2017). Controlled synthesis of water-dispersive and superparamagnetic Fe_3_O_4_ nanomaterials by a microwave-asisted solvothermal method: From nanocrystals to nanoclusters. CrystEngComm.

[30] Rizzuti A., Dissisti M., Mastrorilli P., Sportelli M.C., Cioffi N., Picca R.A., Agostinelli E., Varvaro G., Caliandro R. (2015). Shape-control by microwave-assisted hydrothermal method for the synthesis of magnetite nanoparticles using organic additives. J. Nanopart. Res..

[31] Liang Y.J., Zhang Y., Guo Z., Xie J., Bai T., Zou J., Gu N. (2016). Ultrafast Preparation of Monodisperse Fe_3_O_4_ Nanoparticles by Microwave-Assisted Thermal Decomposition. Chem. Eur. J..

[32] Matijevic E. (1993). Preparation and Properties of Uniform Size Colloids. Chem. Mater..

[33] Li X., Zhang F., Ma C., Saul E., He N. (2012). Green Synthesis of Uniform Magnetite (Fe3O4) Nanoparticles and Micron Cubes. J. Nanosci. Nanotechnol..

[34] Li Q., Kartikowati C.W., Horie S., Ogi T., Iwaki T., Okuyama K. (2017). Correlation between Particle Size/Domain Structure and Magnetic Properties of Highly Crystalline Fe3O4 Nanoparticles. Sci. Rep..

[35] Teja A.S., Koh P.Y. (2009). Synthesis, Properties, and Applications of Magnetic Iron Oxide Nanoparticles. Prog. Cryst. Growth Charact. Mater..

[36] Mascolo M.C., Pei Y., Ring T.A. (2013). Room Temperature Co-Precipitation Synthesis of Magnetite Nanoparticles in a Large pH Window with Different Bases. Materials.

[37] Rahmayanti M. (2020). Synthesisof Magnetite Nanoparticles Using Reverse Co-Precipitation Method With NH_4_OH as Precipitating Agent and Its Stability Test at Various pH. Nat. Sci..

[38] Laurent S., Forge D., Port M., Roch A., Robic C., vander Elst L., Muller R.N. (2008). Magnetic Iron Oxide Nanoparticles: Synthesis, Stabilization, Vectorization, Physicochemical Characterizations and Biological Applications. Chem. Rev..

[39] Castelló J., Gallardo M., Busquets M.A., Estelrich J. (2015). Chitosan (or Alginate)-Coated Iron Oxide Nanoparticles: A Comparative Study. Colloids Surf. A: Physicochem. Eng. Asp..

[40] Harraz F.A. (2008). Polyethylene Glycol-Assisted Hydrothermal Growth of Magnetite Nanowires: Synthesis and Magnetic Properties. Phys. E Low-Dimens. Syst. Nanostructures.

[41] Luo C., Zhang Y., Zeng X., Zeng Y., Wang Y. (2005). The Role of Poly(Ethylene Glycol) in the Formation of Silver Nanoparticles. J. Colloid Interface Sci..

[42] Alibeigi S., Vaezi M.R. (2008). Phase Transformation of Iron Oxide Nanoparticles by Varying the Molar Ratio of Fe^2+^:Fe^3+^. Chem. Eng. Technol..

[43] Testa-Anta M., Ramos-Docampo M.A., Comesaña-Hermo M., Rivas-Murias B., Salgueiriño V. (2019). Raman Spectroscopy to Unravel the Magnetic Properties of Iron Oxide Nanocrystals for Bio-Related Applications. Nanoscale Adv..

[44] Shebanova O.N., Lazor P. (2003). Raman Study of Magnetite (Fe3O4): Laser-Induced Thermal Effects and Oxidation. J. Raman Spectrosc..

[45] Jubb A.M., Allen H.C. (2010). Vibrational Spectroscopic Characterization of Hematite, Maghemite, and Magnetite Thin Films Produced by Vapor Deposition. ACS Appl. Mater. Interfaces.

[46] Li S., Hihara L.H. (2015). A Micro-Raman Spectroscopic Study of Marine Atmospheric Corrosion of Carbon Steel: The Effect of Akaganeite. J. Electrochem. Soc..

[47] Gupta R., Sood A.K., Metcalf P., Honig J.M. (2002). Raman Study of Stoichiometric and Zn-Doped Fe3O4. Phys. Rev. B Condens. Matter.

[48] Nasrazadani S., Raman A. (1993). The Application of Infrared Spectroscopy to the Study of Rust Systems-II. Study of Cation Deficiency in Magnetite (Fe_3_O_4_) Produced During its Transformation to Maghemite (γ-Fe_2_0_3_) And Hematite (α-Fe_2_0_3_). Corros. Sci..

[49] Ishii M., Nakahira M. (1972). Infrared Absorption Spectra and Cation Distributions in (Mn, Fe)_3_O_4_. Solid State Commun..

[50] Stoia M., Istratie R., Păcurariu C. (2016). Investigation of Magnetite Nanoparticles Stability in Air by Thermal Analysis and FTIR Spectroscopy. J. Therm. Anal. Calorim..

[51] Wang Q., Shi J.L., Chen L.D., Yan D.S. (2003). Synthesis of Nanocrystalline Magnetite (Fe_3_O_4_) Films by Self-Reduction Sol-Gel Route. Mater. Sci. Forum.

[52] Namduri H., Nasrazadani S. (2008). Quantitative Analysis of Iron Oxides Using Fourier Transform Infrared Spectrophotometry. Corros. Sci..

[53] Nakahata Y., Borkowski B., Shimoji H., Yamada K., Todaka T., Enokizono M. (2012). Precise Measurement of Magnetization Characteristics in High Pulsed Field. J. Appl. Phys..

[54] Trojanowski S., Ciszek M. (2008). Sensitivity of the Integrating Pulse Magnetometer with a First-order Gradiometer. Rev. Sci. Instr..

[55] Kodama K. (2015). Pulsed-field Magnetometry for Rock Magnetism. Earth Planet Sp..

[56] Kodama K. (2015). Measurement of Dynamic Magnetization Induced by a Pulsed Field: Proposal for a New Rock Magnetism Method. Front. Earth Sci..

[57] Aivazoglou E., Metaxa E., Hristoforou E. (2018). Microwave-Assisted Synthesis of Iron Oxide Nanoparticles in Biocompatible Organic Environment. AIP Adv..

[58] Dehsari H.S., Ksenofontov V., Moller A., Jakob G., Asadi K. (2018). Determining Magnetite/Maghemite Composition and Core−Shell Nanostructure from Magnetization Curve for Iron Oxide Nanoparticles. J. Phys. Chem. C..

[59] Winsett J., Moilanen A., Paudel K., Kamali S., Ding K., Cribb W., Seifu D., Neupane S. (2019). Quantitative Determination of Magnetite and Maghemite in Iron Oxide Nanoparticles Using Mössbauer Spectroscopy. SN Appl. Sci..

[60] Singh A.K., Srivastava O.N., Singh K. (2017). Shape and Size-Dependent Magnetic Properties of Fe_3_O_4_ Nanoparticles Synthesized Using Piperidine. Nanoscale Res. Lett..

[61] LaMer V.K., Dinegar R.H. (1950). Theory, Production and Mechanism of Formation of Monodispersed Hydrosols. J. Am. Chem. Soc..

[62] Smith W.B. (2002). Ethylene glycol to acetaldehyde-dehydration or a concerted mechanism. Tetrahedron.

[63] Skrabalak S.E., Wiley B.J., Kim M., Formo E.V., Xia Y. (2008). On the Polyol Synthesis of Silver Nanostructures: Glycolaldehyde as a Reducing Agent. Nano Lett..

